# EPI-DRC Brasil: When public health campaigns become scientific evidence — a new paradigm for global nephrology

**DOI:** 10.1590/2175-8239-JBN-2026-E010en

**Published:** 2026-07-10

**Authors:** Geraldo Bezerra da Silva, Edison Souza

**Affiliations:** 1 Sociedade Brasileira de Nefrologia, São Paulo, SP, Brazil.; 2 Universidade de Fortaleza, Fortaleza, CE, Brazil.; 3 Universidade do Estado do Rio de Janeiro, Rio de Janeiro, RJ, Brazil.

Chronic kidney disease (CKD) remains a silent and underestimated epidemic, with a global prevalence of around 9.5% (reaching 14% in some regions), despite decades of awareness and prevention efforts^1,2^. In Brazil, the impact of CKD is particularly significant. It is estimated that more than 170,000 patients are currently on dialysis therapy, with continuous growth in prevalence and incidence over the last few years. This scenario is accompanied by marked regional inequalities in access to renal replacement therapy, with substantial differences between high- and low-middle-income regions, reflecting structural disparities in the health system^3^.

Global initiatives such as World Kidney Day (WKD) have played a central role in raising awareness about CKD, being recognized as strategic instruments for public engagement and health mobilization^2,4^. However, despite this ongoing effort, CKD remains largely under-recognized as a global health priority, and current care models still focus predominantly on advanced stages of the disease. In this context, a critical question emerges: to what extent have awareness-based campaigns been able to promote early detection, effective prevention, and, above all, to modify the epidemiological course of CKD?

The EPI-DRC Brasil study offers an innovative answer to this question. By integrating awareness campaigns with structured screening strategies using point-of-care creatinine testing in an unprecedented, large-scale manner, the study transcends the traditional model of educational campaigns and proposes a new paradigm: campaigns as platforms for generating evidence and intervention in public health.

In 2019, in Samoa, an island nation in Polynesia, during the World Kidney Day campaign, a pioneering community program using point-of-care creatinine and HbA1c was implemented, screening 1163 individuals and identifying a CKD prevalence of 44.5%^5^. This value is remarkably similar to that observed in Brazil, where 40.2% of participants presented with a reduced glomerular filtration rate^6^, suggesting that active screening strategies in at-risk populations reveal a substantial hidden burden of kidney disease. In a more recent study in Poland, the frequency of GFR < 60 mL/min/1.73 m^2^ was 2.9%, but the screening was performed in the general population, not only among people with risk factors^7^.

The findings of the EPI-DRC Brasil study are compelling. In a robust cohort of over 8,000 individuals with risk factors, across 20 states and 40 cities in the country, a substantially higher frequency of reduced renal function was identified than that observed in the general population. These data, in themselves, expose a disturbing reality: there is a significant number of individuals potentially living with undiagnosed CKD in Brazil.

Even more relevant is the profile of these individuals. The predominance of intermediate stages (especially G3) suggests a critical window of opportunity for intervention. This is a clinical inflection point where therapeutic strategies, both pharmacological and non-pharmacological, can effectively modify the trajectory of the disease^8^. In this context, the study reinforces a central message: the problem is not only the prevalence of CKD but also the delay in diagnosis.

Another particularly worrying finding is the low level of knowledge among the population. In the EPI-DRC Brasil, only 35.2% of participants reported recognizing risk factors for CKD^6^. These data are consistent with previous Brazilian evidence: in a population-based study conducted in Fortaleza, only 17.2% of individuals knew how to correctly define CKD, and only 5.8% understood the meaning of creatinine^9^. Taken together, these findings reveal a critical gap between exposure to campaigns and effective health literacy, suggesting that disseminated information does not necessarily translate into assimilated knowledge. International studies reinforce this limitation, demonstrating low levels of knowledge even in urban populations with access to information^10^.

The study also highlights significant regional heterogeneity, with marked variations in the frequency of reduced renal function among Brazilian states. These differences likely reflect not only epidemiological variations but also structural inequalities in access to care, diagnosis, and health education^3^.

From a methodological and operational standpoint, the EPI-DRC Brasil study unequivocally demonstrates the viability of using point-of-care technologies in population-based strategies. In a country of continental dimensions, with limitations in laboratory infrastructure in several regions, this approach represents a strategic advancement. More than a technological innovation, it is an innovation in the care model, now reinforced by converging evidence from different global contexts.

Naturally, limitations must be acknowledged. The cross-sectional nature of the study and the use of a single creatinine measurement preclude a definitive diagnosis of CKD. However, this limitation does not diminish the value of the findings; on the contrary, it reinforces the role of screening as a gateway to diagnostic confirmation and longitudinal care.

Perhaps the greatest merit of EPI-DRC Brasil lies in its conception: a coordinated, national effort involving multiple centers and professionals, capable of transforming a health campaign into a platform for applied science. This level of collective engagement is, in itself, a milestone for Brazilian nephrology.

More than just a study, the EPI-DRC Brasil study proposes a paradigm shift. It suggests that tackling CKD will not depend exclusively on new therapies or biomarkers, but also on the ability to integrate science, technology, and collective action into effective public health strategies.

In a global context where the burden of CKD continues to grow, especially in low- and middleincome countries^1^, the Brazilian experience offers a replicable model. Transforming campaigns into science and science into care may be one of the most promising paths to altering the course of this silent epidemic.

## Data Availability

The data supporting the findings of this study (EPI-DRC Brasil) are available from the corresponding author upon request, respecting applicable ethical and confidentiality standards.
