# Supplementary material for: EPI-DRC Brasil: When public health campaigns become scientific evidence — a new paradigm for global nephrology
Source: J Bras Nefrol. 2026 Jul 10;48(3):e2026E010. doi: 10.1590/2175-8239-JBN-2026-E010en (PMC13366617; doi:10.1590/2175-8239-JBN-2026-E010en)
Supplement: Translation

# Translation: EPI-DRC Brasil: quando campanhas de saúde pública se transformam em evidência científica — um novo paradigma para a nefrologia global

**DOI:** 10.1590/2175-8239-JBN-2026-E010pt

**Authors:** Geraldo Bezerra da Silva, Edison Souza

**Affiliations:** 1 Sociedade Brasileira de Nefrologia, São Paulo, SP, Brasil.; 2 Universidade de Fortaleza, Fortaleza, CE, Brasil.; 3 Universidade do Estado do Rio de Janeiro, Rio de Janeiro, RJ, Brasil.

A doença renal crônica (DRC) permanece uma epidemia silenciosa e subestimada, com prevalência global em torno de 9,5% (chegando a 14% em algumas regiões), apesar de décadas de esforços em conscientização e prevenção^1,2^. No Brasil, o impacto da DRC é particularmente expressivo. Estima-se que mais de 170 mil pacientes estejam atualmente em terapia dialítica, com crescimento contínuo da prevalência e da incidência ao longo dos últimos anos. Esse cenário é acompanhado por marcadas desigualdades regionais no acesso à terapia renal substitutiva, com diferenças substanciais entre regiões mais e menos desenvolvidas, refletindo disparidades estruturais no sistema de saúde^3^.

Iniciativas globais como o Dia Mundial do Rim (DMR) têm desempenhado papel central na promoção da conscientização sobre a DRC, sendo reconhecidas como instrumentos estratégicos para engajamento público e mobilização em saúde^2,4^. No entanto, apesar desse esforço contínuo, a DRC permanece amplamente sub-reconhecida como prioridade global de saúde, e os modelos atuais de cuidado ainda se concentram predominantemente em estágios avançados da doença. Nesse contexto, emerge uma questão crítica: até que ponto campanhas baseadas em conscientização têm sido capazes de promover detecção precoce, prevenção efetiva e, sobretudo, de modificar o curso epidemiológico da DRC?

O estudo EPI-DRC Brasil oferece uma resposta inovadora a essa pergunta. Ao integrar, de forma inédita e em larga escala, ações de conscientização com estratégias estruturadas de rastreamento utilizando o teste *point-of-care* de creatinina, o estudo transcende o modelo tradicional de campanhas educativas e propõe um novo paradigma: campanhas como plataformas de geração de evidência e intervenção em saúde pública.

Em 2019, em Samoa, país insular da Polinésia, durante a campanha do Dia Mundial do Rim, foi implementado, de forma pioneira, um programa comunitário com uso de creatinina *point-of-care* e HbA1c, rastreando 1163 indivíduos e identificando uma prevalência de DRC de 44,5%^5^. Esse valor é notavelmente semelhante ao observado no Brasil, onde 40,2% dos participantes apresentaram taxa de filtração glomerular reduzida^6^, sugerindo que estratégias ativas de rastreamento em populações de risco revelam uma carga oculta substancial de doença renal. Em estudo mais recente, na Polônia, a frequência de TFG < 60mL/min/1,73 m^2^ foi de 2,9%; porém, o rastreamento foi realizado na população geral, não apenas entre pessoas com fatores de risco^7^.

Os achados do EPI-DRC Brasil são contundentes. Em uma coorte robusta de mais de 8.000 indivíduos com fatores de risco, em 20 estados do país e 40 cidades, identificou-se uma frequência de função renal reduzida substancialmente superior à observada na população geral. Esse dado, por si só, expõe uma realidade inquietante: há um contingente expressivo de indivíduos potencialmente com DRC não diagnosticada no Brasil.

Mais relevante ainda é o perfil desses indivíduos. A predominância de estágios intermediários (especialmente G3) sugere uma janela crítica de oportunidade para intervenção. Trata-se de um ponto de inflexão clínico onde estratégias terapêuticas, farmacológicas e não farmacológicas podem efetivamente modificar a trajetória da doença^8^. Nesse contexto, o estudo reforça uma mensagem central: o problema não é apenas a prevalência da DRC, mas também o atraso no diagnóstico.

Outro achado particularmente preocupante é o baixo nível de conhecimento da população. No EPI-DRC Brasil, apenas 35,2% dos participantes relataram reconhecer fatores de risco para DRC^6^. Esse dado é consistente com evidências brasileiras prévias: em um estudo de base populacional realizado em Fortaleza, apenas 17,2% dos indivíduos sabiam corretamente definir DRC, e apenas 5,8% compreendiam o significado da creatinina^9^. Em conjunto, esses achados revelam uma lacuna crítica entre exposição a campanhas e efetiva literacia em saúde, sugerindo que a informação disseminada não necessariamente se traduz em conhecimento assimilado. Estudos internacionais reforçam essa limitação, demonstrando baixos níveis de conhecimento mesmo em populações urbanas e com acesso à informação^10^.

O estudo também evidencia heterogeneidade regional significativa, com variações marcantes na frequência de função renal reduzida entre os estados brasileiros. Essas diferenças provavelmente refletem não apenas variações epidemiológicas, mas também desigualdades estruturais no acesso ao cuidado, ao diagnóstico e à educação em saúde^3^.

Do ponto de vista metodológico e operacional, o EPI-DRC Brasil demonstra, de forma inequívoca, a viabilidade do uso de tecnologias *point-of-care* em estratégias populacionais. Em um país de dimensões continentais, com limitações de infraestrutura laboratorial em diversas regiões, essa abordagem representa um avanço estratégico. Mais do que uma inovação tecnológica, trata-se de uma inovação no modelo de cuidado, agora reforçada por evidências convergentes de diferentes contextos globais.

Naturalmente, limitações devem ser reconhecidas. A natureza transversal do estudo e o uso de uma única medida de creatinina impedem o diagnóstico definitivo de DRC. No entanto, essa limitação não diminui o valor dos achados; ao contrário, reforça o papel do rastreamento como porta de entrada para confirmação diagnóstica e cuidado longitudinal.

O maior mérito do EPI-DRC Brasil talvez resida em sua concepção: um esforço coordenado, nacional, envolvendo múltiplos centros e profissionais, capaz de transformar uma campanha de saúde em uma plataforma de ciência aplicada. Esse nível de engajamento coletivo é, por si só, um marco para a nefrologia brasileira.

Mais do que um estudo, o EPI-DRC Brasil propõe uma mudança de paradigma. Ele sugere que o enfrentamento da DRC não dependerá exclusivamente de novas terapias ou biomarcadores, mas também da capacidade de integrar ciência, tecnologia e ação coletiva em estratégias de saúde pública efetivas.

Em um cenário global onde a carga da DRC continua a crescer, especialmente em países de baixa e média renda^1^, a experiência brasileira oferece um modelo replicável. Transformar campanhas em ciência e ciência em cuidado pode ser um dos caminhos mais promissores para alterar o curso dessa epidemia silenciosa.

## Data Availability

Os dados que sustentam os achados deste estudo (EPI-DRC Brasil) estão disponíveis com o autor correspondente, mediante solicitação, respeitando as normas éticas e de confidencialidade aplicáveis.
